# Negro Dentists

**Published:** 1889-06

**Authors:** 


### EDITORIALS AND MISCELLANEOUS.

NEGRO DENTISTS.—There are but two colored medical colleges in the United States. The Meharry Medical College is at Nashville, Tenn., and at its recent commencement graduated fifteen.

The school has a dental department and graduated six in Dentistry.

The usual addresses were made on the occasion. A salutatory was delivered by one of the sable orators, which we publish as a specimen of graudiloquence and rhetoric which is in vogue at this school. We suppose it was written for him by some young Sophomore in the Vanderbilt University:

"From the defiles of indifference with its dismal swamps of poverty, between the mountains of prejudices and opposition, a little band of Spartan heroes from Mehary's walls are endeavoring to beat back merciless disease, voracious death, and to bridge the waters of oblivion. The echo of their words, stern and unconditional, is wafted back—"Vestigia nulla retrosum." No steps backward. Our eyes watch their progress with pride, our hearts leap with impetuous ambition at the thought of swelling their ranks and sounding the onset with our own war-cry, "Niam inveni aut unam fac." Through the labyrinth of life, with its multitudes of difficulties and obstructions, it shall ever be the motto of the class of '89 to "find or make one."

Friends, to-night you have come from different parts of your beautiful city, the Athens of the South, with flowers, congratulations and other substantial souvenirs of regard and esteem to bedeck our entrance into professional life. Our stay has been so pleasant among you that your images wreathed in your many acts of kindness shall ever be fondly hung in the brightest halls of memory.

To our illustrious guests with us to-night we would say, we feel honored with your presence.

To you, one and all, in the name of the faculty whose untiring efforts have prepared us for our work; in the name of those great philanthropists of North and South whose open purse has never been shut to the needs of our school; in the name of that noble-hearted family, Mehary, who in the founding of this institution whose name it bears, unconsciously erected to themselves a monument which will last when they, and massive spires of granite shall yield to the insatiate tooth of time; and finally, in the name of the class of '89, do we bid you a hearty welcome.

